# Association between *MMP3* and *TIMP3* polymorphisms and risk of osteoarthritis

**DOI:** 10.18632/oncotarget.18745

**Published:** 2017-06-27

**Authors:** Zhichao Tong, Yang Liu, Bo Chen, Liang Yan, Dingjun Hao

**Affiliations:** ^1^ Department of Bone Diesase and Bone Tumor, Hong Hui Hospital, Xi’an Jiaotong University College of Medicine, Shaanxi 710054, China; ^2^ Department of Orthopaedics, Hong Hui Hospital, Xi’an Jiaotong University College of Medicine, Shaanxi 710054, China; ^3^ Department of Spinal Surgery, Hong Hui Hospital, Xi’an Jiaotong University College of Medicine, Shaanxi 710054, China

**Keywords:** MMP-3, TIPM-3, osteoarthritis, polymorphisms

## Abstract

Osteoarthritis (OA) is the most commonly occurring degenerative joint disease worldwide, and its incidence has increased in recent years. We evaluated whether there is the association between *MMP-3* and *TIMP-3* variants and susceptibility to OA in a Chinese population. Venous blood samples were collected from 431 female participants (200 cases and 231 controls) at Hong Hui Hospital, Xi’an Jiaotong University College of Medicine between 2015 and 2016. After genotyping the samples using standard protocols, the association between *MMP-3* and *TIMP-3* single nucleotide polymorphisms and risk of OA was assessed by calculating odds ratios (ORs) and 95% confidence intervals (95% CIs) using unconditional logistic regression analysis. The minor G allele of rs650108 was associated with OA risk in a recessive model (*p* = 0.034, OR = 1.82, 95%CI = 1.04-3.18), while the minor A allele of rs715572 was associated with OA risk in a recessive model (*p =* 0.030, OR = 1.88, 95%CI = 1.05-3.34). Thus a suggestive association was observed in a discovery case-control study between OA and two common SNPs, rs650108 in *MMP-3* and rs715572 in *TIMP-3*.

## INTRODUCTION

Osteoarthritis (OA) is the most commonly occurring degenerative joint disease worldwide [1]. Affecting primarily middle-aged and older individuals, its incidence has been increasing in recent years. OA patients (especially older adults) are characterized by narrowing of the joint line in such joints as the hip joint, knee or small joints of the hand, foot, and spine, accompanied with progressively greater loads on the subchondral bone, which results in late-onset articular cartilage cataplasia, joint pain and joint stiffness [2, 3]. Previous epidemiological findings suggest that one-third of elderly adults (≥ 65) suffer from OA. In addition, several recent studies have shown that both environmental and genetic factors contribute to OA [4, 5].

Much of our current knowledge about OA has come from molecular genetic investigations [6, 7]. Genome-wide association studies have demonstrated that OA susceptibility is influenced by genetic predisposition. When we searched for susceptibility loci for OA, it was apparent that many loci have particular relevance for the development of OA at particular skeletal sites, and many of the linkages to OA differed depending on the ethnicity of the patient [8–10]. A search of the U. S. National Library of Medicine database (MEDLINE) (http://www.ncbi.nlm.nih.gov/IEB/Research/Acembly/av.cgi) for genetic information focusing on the relationship between genetic variation and disease reported that matrix metalloproteinase-3 gene (*MMP-3*) and the tissue inhibitors of matrix metalloproteinases-3 gene (*TIMP3)* associated with rheumatoid arthritis and arthritis. Putative interactions between TIMP-3 and MMP-3 proteins were also reported [11–13]. *MMP-3* and *TIMP-3* have also been shown to be involved in a variety of other ailments, including prostate cancer, ulcerative colitis and abdominal aortic aneurysm [14–16]. Moreover, compelling evidence suggests that *MMP3* and *TIMP-3* polymorphisms may contribute to OA [17, 18].

MMPs are a large family of extracellular zinc-dependent endopeptidases that catalyze the production and degradation of extracellular matrix (ECM) under both physiological and pathological conditions, while TIMPs function as MMP inhibitors [19, 20]. Consequently, TIMPs act as key regulators of bone metabolism [17, 21]. One previous study reported *TIMP-3* is expressed in osteoblasts, and that its expression is up-regulated during differentiation of MC3T3-E1 cells. In addition, the expression of osteocalcia, osteonectin, and osteopontin are all inhibited by overexpression of *TIMP-3* [22]. Hence, MMP-3 and TIMP-3 are important mediators for osteoblast function.

In the present study, we investigated whether allelic variants of *MMP-3* and *TIMP-3* associate with OA. We selected 12 single nucleotide polymorphisms (SNPs) and performed a comprehensive association analysis with OA. Our risk analysis revealed a suggestive association between OA and two common SNPs.

## RESULTS

In total, 431 female subjects were genotyped (200 cases and 231 healthy controls) for 12 SNPs. The mean age of the cases was significantly greater than that of the controls (59.3 vs. 54.0 years, *p* < 0.001) (Table 1).

**Table 1:** Characteristics of cases and controls in this study

Variable	Normal(n=231)	OA(n=200)	P-value
Age(SD), year	54.0(9.3)	59.3(3.6)	<0.001
Female	231	200	-

Table 2 summarizes the MAF, Hardy-Weinberg equilibrium *p* values, and Pearson's χ^2^ *p* values for the 12 SNPs examined in this study population. Although no SNPs were outside the HWE (*p* < 0.05), Pearson's χ^2^ analysis revealed that 4 SNPs were not associated with OA (*p* < 0.05 for both).

**Table 2:** Allele frequencies in cases and controls and odds ratio estimates for osteoarthritis

Gene	rs number	Role	allele(A^1^/B)	Major allelic frequency		HWE *P* value^2^	OR(95%CI)	P-value
				case	control			
MMP3	rs639752	Intron	C/A	0.33	0.31	0.09	1.10(0.82-1.46)	0.53
	rs650108	Intron	G/A	0.41	0.37	0.07	1.20(0.91-1.57)	0.20
	rs520540	Coding exon	A/G	0.33	0.31	0.09	1.10(0.82-1.46)	0.53
	rs646910	Intron	A/T	0.08	0.06	0.15	1.41(0.82-2.42)	0.21
	rs602128	Coding exon	A/G	0.33	0.30	0.21	1.14(0.85-1.52)	0.38
	rs679620	Coding exon	T/C	0.33	0.31	0.13	1.10(0.82-1.45)	0.57
	rs678815	Intron	G/C	0.32	0.31	0.13	1.04(0.78-1.40)	0.79
	rs522616	Promoter	C/T	0.41	0.41	0.06	1.00(0.76-1.32)	0.98
TIMP3	rs715572	Intron	A/G	0.38	0.32	0.65	1.30(0.99-1.72)	0.06
	rs8136803	Intron	T/G	0.04	0.05	1.00	0.83(0.43-1.60)	0.59
	rs9609643	Intron	A/G	0.13	0.15	0.61	0.85(0.58-1.25)	0.41
	rs11547635	Coding exon	T/C	0.33	0.37	0.07	0.84(0.63-1.11)	0.21

^1^Minor allele; ^2^Site with HWE P ≤ 0.05 excluded. HWE: Hardy-Weinberg equilibrium.

Table 3 lists the associations between the *MMP-3* and *TIMP-3* SNPs and OA risk in co-dominant, dominant, and recessive models. The minor G allele of rs650108 in *MMP-3* was associated with OA risk in the recessive model (*p =* 0.034, OR = 1.82, 95%CI = 1.04-3.18), while the minor A allele of rs715572 in *TIMP-3* was associated with OA risk in the recessive model (*p =* 0.03, OR = 1.88, 95%CI = 1.05-3.34).

**Table 3:** Logistic regression analysis of the association between single-nucleotide polymorphisms and osteoarthritis risk n (%)

SNP	Model	Genotype	Group=control	Group=case	OR (95% CI)	P-value	AIC	BIC
rs650108	Codominant	A/A	86 (37.4%)	72 (36%)	1	0.098	595.4	607.6
		A/G	120 (52.2%)	93 (46.5%)	0.93 (0.61-1.40)			
		G/G	24 (10.4%)	35 (17.5%)	1.74 (0.95-3.19)			
	Dominant	A/A	86 (37.4%)	72 (36%)	1	0.770	597.9	606.1
		A/G-G/G	144 (62.6%)	128 (64%)	1.06 (0.72-1.57)			
	Recessive	A/A-A/G	206 (89.6%)	165 (82.5%)	1	0.034	593.5	601.6
		G/G	24 (10.4%)	35 (17.5%)	1.82 (1.04-3.18)			
rs715572	Codominant	G/G	104 (45%)	80 (40%)	1	0.090	596.4	608.6
		G/A	105 (45.5%)	87 (43.5%)	1.08 (0.72-1.62)			
		A/A	22 (9.5%)	33 (16.5%)	1.95 (1.06-3.60)			
	Dominant	G/G	104 (45%)	80 (40%)	1	0.290	598.2	606.3
		G/A-A/A	127 (55%)	120 (60%)	1.23 (0.84-1.80)			
	Recessive	G/G-G/A	209 (90.5%)	167 (83.5%)	1	0.030	594.6	602.7
		A/A	22 (9.5%)	33 (16.5%)	1.88 (1.05-3.34)			

AIC: Akaike's information criterion; BIC: Bayesian information criterion; SNP: Single-nucleotide polymorphism.

## DISCUSSION

MMP-3 is a zinc-dependent endopeptidase that catalyzes the degradation of various collagenous, ECM and non-collagenous basement membrane proteins [23]. In addition, MMP-3 cleaves the propeptide unit to activate other MMPs. Consequently, the MMP-3 substrate profile crucially affects matrix degradation and remodeling within normal and diseased musculoskeletal soft tissues [23, 24]. Our results showed that there is a weak association between *MMP-3* SNPs and OA susceptibility. Although these gene variants were previously associated with cartilage and tendon pathology risk, no association between *MMP-3* SNPs and the degeneration of knee articular cartilage or patellar tendon have been reported [25].

TIMP-3 is TIMP family member that displays unique molecular properties and features. For example, TIMP-3 appears able to induce apoptosis in melanoma cells, retinal pigment epithelial cells, and breast cancer cells [26–28]. TIMP-3 also acts as a local cytokine in bone, regulating bone metabolism by suppressing osteoblast differentiation and inducing osteoblast apoptosis [29, 30]. In addition, *TIMP-3* variants were weakly associated with OA susceptibility in our study.

The participants in the present study were genetically unrelated native residents in Xi’an, Shaanxi Province. All individuals were in Hardy-Weinberg equilibrium and were ethnically homogeneous, which made confounding ethnic heterogeneity less likely. The likely reasons some previous studies did not detect significant evidence of an association between *MMP-3* or *TIMP-3* SNPs and susceptibility to OA are that (a) the studies were limited by the small sample size or (b) there was heterogeneity among the study populations further complicated by the allelic frequencies in the various susceptibility genes [31].

There is functional evidence for the role of *MMP-3* in OA, which makes it an interesting study target, even though this gene is not the main *MMP* related to OA. MMP-3 is the main MMP family member involved in cartilage degradation, and is thought to have broad substrate specificity enabling to be active against types II, III, and IV collagens, laminin, proteoglycans, and fibronectin. Moreover, MMP-3 is able to activate MMP-1, MMP-2, MMP-9 and MMP-13 [32, 33]. An earlier study reported that levels of MMP-3 in the synovial fluid and serum are elevated in canine models of OA [34, 35]. In addition, the major histocompatibility complex (MHC) can present the fragments generated through MMP-3-catalyzed collagen hydrolysis to T cells, and promote the activation and release of inflammatory cytokines, which in turn increase *MMP-3* expression in cartilage cells and synovial fibroblasts. These processes result in increased collagenase activity and aggravation of joint inflammation [36]. Similarly, functional evidence for the role of *TIMP-3* in OA also makes it an interesting study target for OA. TIMP-3 is reported to be highly expressed in the endosteal region of bone marrow, particularly by osteoblasts, and to regulate hematopoietic stem cell proliferation (HSC), differentiation and trafficking *in vivo*, as well as bone turnover. TIMP-3 is also expressed by stromal cells forming HSC niches within the bone marrow. TIMP-3 may thus be involved in regulating both hematopoiesis and bone remodeling.

## CONCLUSIONS

The present case-control study revealed a suggestive association between OA and two common SNPs, rs650108 and rs715572, in *MMP-3* and *TIMP-3*, respectively. Both of these genes were previously reported to have a functional role in OA but the finding was not replicated with statistical significance in follow-up analyses of OA.

## MATERIALS AND METHODS

### Ethics statement

The study was approved by the Ethics Committee of Hong Hui Hospital, Xi’an Jiaotong University College of Medicine. An investigative agreement and a standard statistical survey questionnaire were drafted which followed the World Medical Association Declaration of Helsinki. The primary protocols were explained to each participant before they signed an informed consent.

### Subjects

Venous blood samples were collected from 431 female participants (200 cases and 231 controls) at the Orthopedics and Medical Examination Center at Hong Hui Hospital, Xi’an Jiaotong University College of Medicine between 2015 and 2016. All participants were native residents in Xi’an, Shaanxi Province, and all were genetically unrelated. We diagnosed OA based on the American College of Rheumatology criteria. These included primary OA with any symptoms in one or two knees and radiographic signs of OA graded as ≥ 2 on the Kellgren-Lawrence (K/L) grading system. We excluded the controls with post-traumatic or post-septic arthritis, skeletal or developmental aldysplasia, and inflammatory arthritis. The closing date of eligibility was June 31, 2016, at which time we have enough patients with OA for subsequent follow-up.

### Epidemiological and clinical data

The questionnaire included age, sex, and profession. The data from the standard statistical survey questionnaire were logged in Microsoft Excel 2010 software. The 431 blood samples were stored in accordance with unified standards (-80°C). We collected clinical information from medical records and radiological diagnoses.

### SNP selection and genotyping

Twelve loci from *MMP-3* and *TIMP-3* were randomly selected for our study. Minor allele frequencies (MAF) of 4 loci were greater than 5% in the HapMap of the Chinese Han Beijing population. DNA was extracted from 5 ml of whole EDTA-blood using the phenol-chloroform extraction method and was quantified using high performance liquid chromatography (NanoDrop2000; Thermo Fisher Scientific, Waltham, MA, USA) [37]. The DNA was then stored at -20°C before genotyping. Sequenom MassARRAY Assay Design 3.0 Software was used to design the multiplexed SNP MassEXTENDED assay (Sequenom, Inc., San Diego, CA, USA). Genotyping was done according to the standard protocol recommended by the manufacturer. Table 4 shows the primers used for genotyping the 12 loci from *MMP-3* and *TIMP-3*. SequenomTyper 4.0 software was used for the data analysis [38].

**Table 4:** Primers used for this study

SNP_ID	1st-PCRP	2nd-PCRP	UEP_SEQ
rs639752	ACGTTGGATGCAGAT AAATTCTCCACTTGC	ACGTTGGATGGGCTGCA ATGCAGGGAAAAG	tGGGAAGAAAGAAATAGGTGAT
rs650108	ACGTTGGATGGTCA CTGTCTCATTGTGTGT	ACGTTGGATGTCAGGT AGAGGTGACAAGTG	tAAGTGGGTGAGGTTAGA
rs520540	ACGTTGGATGGCGAA AGGGCTTAACTGTTAT	ACGTTGGATGCCAGCT CGTACCTCATTTCC	CTCGTACCTCATTTCCTCTGAT
rs646910	ACGTTGGATGCCAC TGTAAGCTGGTGACTA	ACGTTGGATGGTTA AGCCCTTTCGCTTTAG	CGCTTTAGAAATACACTTTAGCATCT
rs602128	ACGTTGGATGCTTC GGGATGCCAGGAAA	ACGTTGGATGAAGCT GGACTCCGACACTCT	CAGGTGTGGAGTTCCTGA
rs679620	ACGTTGGATGAACAGG ACCACTGTCCTTTC	ACGTTGGATGAGA AATATCTAGAAAACTAC	tcTCTAGAAAACTACTACGACCTC
rs678815	ACGTTGGATGAATGCA ACGTAATTTTAGC	ACGTTGGATGTGGA GTATTTCTCTAGCTTG	TCTCTAGCTTGCTGAAATAATG
rs522616	ACGTTGGATGCGTAG CTGCTCCATAAATAG	ACGTTGGATGACAGA GAGAATTTCAGTCCG	gaCGGTAAGCAATGTAATTCATTTCA
rs715572	ACGTTGGATGTAGTGA GTGTCCAAGGAACC	ACGTTGGATGAA GCTAGTCCACCTCTCTTC	CTCTCTTTCTTCCAGCA
rs8136803	ACGTTGGATGTGGCAC ATAACAGGCACCTC	ACGTTGGATGCTG TGTGTGGCACTTTATAC	TGGCACTTTATACAAGAAATCACAC
rs9609643	ACGTTGGATGTGAA GAGATGTCTGGCTTTG	ACGTTGGATGGTCCC AAGGGTTTATAATAG	ATAGGAAAATGCCTCTACTTTA
rs11547635	ACGTTGGATGTGCC CCATGTGCAGTACATC	ACGTTGGATGACTG GTACTTGTTGACCTCC	tGCTTAAGGCCACAGAGACTCTC

### Statistical analysis

Microsoft Excel 2010 software and SPSS 20.0 statistical software were for the statistical analysis of the data, including Hardy-Weinberg equilibrium analysis of the control group and χ^2^ test. All *p* values were two-sided. The association between the risk of OA and the genotypes was assessed by calculating odds ratios (ORs) and 95% confidence intervals (95% CIs) using unconditional logistic regression analysis. The statistical results of this study were reviewed by Fengjiao Wang from the Xi'an Tiangen Precision Medical Institute.
